# Oral Administration of *Sargassum horneri* Improves the HDM/DNCB-Induced Atopic Dermatitis in NC/Nga Mice

**DOI:** 10.3390/nu12082482

**Published:** 2020-08-18

**Authors:** Eui Jeong Han, Ilekuttige Priyan Shanura Fernando, Hyun-Soo Kim, You-Jin Jeon, Dissanayaka Mudiyanselage Dinesh Madusanka, Mawalle Kankanamge Hasitha Madhawa Dias, Youngheun Jee, Ginnae Ahn

**Affiliations:** 1 Department of Food Technology and Nutrition, Chonnam National University, Yeosu 59626, Korea; iosu5772@naver.com (E.J.H.); dmdmadusanka88@gmail.com (D.M.D.M.); hasithadiasm17636@gmail.com (M.K.H.M.D.); 2 Department of Marine Bio-Food Sciences, Chonnam National University, Yeosu 59626, Korea; shanurabru@gmail.com; 3 National Marine Biodiversity Institute of Korea, 75, Jangsan-ro 101 gil, Janghang-eup, Seocheon 33662, Korea; gustn783@naver.com; 4 Department of Marine Life Science, School of Marine Biomedical Sciences, Jeju National University, Jeju 63243, Korea; youjin2014@gmail.com; 5 Department of Veterinary Medicine and Veterinary Medical Research Institute, Jeju National University, Jeju 63243, Korea; yhjee@jejunu.ac.kr

**Keywords:** *Sargassum horneri*, atopic dermatitis, house dust mite (HDM), 2,4-Dinitrochlorobenzene (DNCB)

## Abstract

The present study investigated the protective effects of *Sargassum horneri* (*S. horneri*) ethanol extract (SHE) against atopic dermatitis (AD), known as an abnormal immune response in house dust mite (HDM)/2,4-dinitrochlorobenzene (DNCB)-stimulated NC/Nga mice. The oral administration of SHE attenuated the AD symptoms, including the skin dermatitis severity, transepidermal water loss (TEWL), and ear edema in HDM/DNCB-stimulated mice. Moreover, the histological analysis revealed that SHE improved epidermal hyperplasia and hyperkeratosis, and reduced the dermal infiltrations of mast cells and eosinophils. Moreover, SHE downregulated the expression levels of cytokines (interleukin (IL)-6, IL-10, and interferon (IFN)-γ) and chemokines (Regulated on Activation, Normal T Cell Expressed and Secreted (RANTES), Eotaxin, and Thymus and activation-regulated chemokine (TARC)) by decreasing the expression levels of atopic initiators (IL-25 and IL-33) in HDM/DNCB-stimulated skin. The oral administration of SHE decreased the spleen size, reducing expression levels of AD-related cytokines (IL-4, IL-5, IL-6, IL-10, IL-13, IFN-γ, and TARC) by regulating the expressions of Tbx21 (T-bet), GATA Binding Protein 3 (GATA-3), and Signal transducer and activator of transcription 3 (STAT3). Moreover, SHE significantly attenuated the serum immunoglobulin (Ig)G_1_ and IgG_2a_ levels in HDM/DNCB-stimulated mice. Collectively, these results suggest that *S. horneri* could be an ingredient of functional food against abnormal immune response.

## 1. Introduction

*Sargassum horneri* (*S. horneri*) is an edible brown seaweed, distributed along the coastal areas of South Korea, China, and Japan. Recently, the Ministry of Food and Drug Safety of South Korea (KMFDS) declared *S. horneri* to be a food material, based on recent studies that indicate numerous biological activities like antioxidant, anti-inflammatory, and immunomodulatory effects [1,2,3]. In vitro and in vivo evaluations indicate that active components purified from *S. horneri*, such as sulfated polysaccharides, sagachromanol, mojabanchromanol, loliolide, and apo-9 fucoxanthinone, possess antioxidant, anti-allergic, anti-inflammatory, and immune-modulatory effects [4]. According to recent studies, the ethanol extract of *S. horneri* is rich in mojabanchromanol and loliolide, which show anti-allergic and anti-inflammatory effects [3,5].

Functional foods beneficially affect one or more target functions in the body, beyond nutritional effects, in a way that is relevant to either the state of wellbeing and health, or the reduction of the risk of disease [6]. The topic of functional foods has acquired a rapid growth of interest with recent changes in the human lifestyle, such as improved living standards, rapid industrialization, and increased elderly population. Recent research has exploited the roles of natural materials like plants and seaweed functional foods that contribute to enhancing human health in various aspects, like improvement of liver function, sleep, and diabetes [7,8,9]. However, anthropogenic activities that contribute to the increase of harmful environmental factors like fine dust, heavy metal, microplastics, etc., caused a recent increase in abnormal cutaneous immune diseases, including atopic dermatitis (AD), asthma, immune deficiency, and immune overactivation [10]. AD is the most commonly observed abnormal immune disease characterized by erythema, hemorrhage, edema, excoriation/erosion, scarring/dryness, and itching [11]. There are two major forms of AD, one in which the disease is triggered by allergens with potential immunoglobulin E (IgE) dependency, and one in which the disease appears to be IgE independent [12]. According to the instruction of Korea Ministry of Food and Drug Safety (KMFDS), natural foods contribute to remedying or improving AD symptoms that can be used as a functional food.

Although numerous bioactive natural products of *S. horneri* were studied, little information is available on the potential of *S. horneri* for remediating hypersensitive immune responses like AD and its underlying mechanisms. The present study for the first time evaluates the effects of *S. horneri* ethanolic extract (SHE) on the abnormal hypersensitive immune responses in human dust mite (HDM)/2,4-dinitrochlorobenzene (DNCB)-induced mice model, and its value as a functional food material.

## 2. Materials and Methods

### 2.1. Chemicals and Sample

House dust mite extract ointment was purchased from Biostir Inc. (Biostir AD, Kobe, Japan). Positive control *Lactobacillus plantarum* CJLP 133, a Korean FDA approved product, was purchased from CJ CheilJedang CORP (Seoul, South Korea). Dinitrochlorobenzene (DNCB) and sodium dodecyl sulfate (SDS), phosphate-buffered saline (PBS), formalin, and acetone were purchased from Sigma Chemical Co. (St, Louis, Mo, USA). IgG_1_ and IgG_2a_ ELISA kits were purchased from Bethyl Laboratories (Montgomery, TX, USA). Trizol reagent was purchased from the Molecular Research Center (Montgomery, OH, USA). cDNA synthesis kit was purchased from Promega Co. (‎Madison, WI, USA). Other chemicals and reagents used were the highest grades available commercially. The SHE used in this study was the same as the one used in previous studies [13,14,15].

### 2.2. Mice

NC/Nga female mice (8 weeks old), reared under specific pathogen-free conditions, were purchased from Orient Bio (Gwangju, Korea). The mice were housed under the conditions following; a constant temperature of 23 ± 1.5 °C, a humidity of 55 ± 15%, and lighting followed the 12 h on/ 12 h off-cycle. Food (5L79, Orient Bio, Seongnam, Korea) and tap water were provided ad libitum for all mice, and clean litter (BETA CHIP, Orient Bio) was used during the experiments. All mice procedures were approved by the Institutional Animal Care and Use Committee of the Chonnam National University (No.CNU IACUC-YS-2016-6).

### 2.3. Induction of AD and Oral Administration of SHE

**Figure 1:**
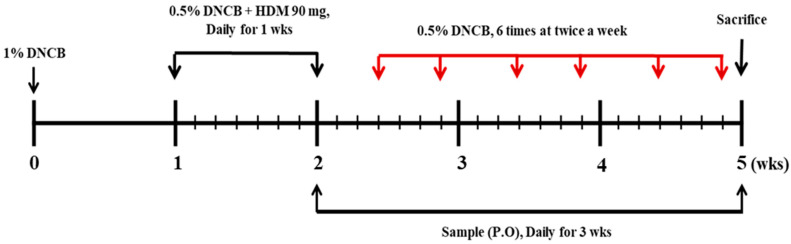
Suppression of HDM/DNCB-induced AD by oral administration of SHE (10, 50, and 100 mg/kg). Schematic illustration of 6-week experimental design.

For the disruption of the skin barrier, the dorsal hair of the mice was shaved using an electronic shaver and hair removal cream, and applied with 150 µL of 4% SDS, before 3 h of DNCB and HDM (AD cream, Biostir-AD, Biostir, Kobe, Japan) application. Then, DNCB and HDM were applied to the dorsal skin for the AD induction, according to the approach indicated in Figure 1. At 14 days of AD induction, the mice were randomized into six groups, as follows—naïve group (control, *n* = 8), AD group (HDM/DNCB applied mice, *n* = 8), SHE 10 group (HDM/DNCB and SHE 10 mg/kg co-applied mice, *n* = 8), SHE 50 group (HDM/DNCB and SHE 50 mg/kg co-applied mice, *n* = 8), SHE 100 groups (HDM/DNCB and SHE 100 mg/kg co-applied mice, *n* = 8), and a P.C group (a positive control group with HDM/DNCB and CJLP 133 800 mg/kg co-applied mice, *n* = 8). SHE was oral administrated to mice using an oral-zoned needle connected to a 1 mL syringe. On the 35th day, the mice were dissected after the measurement of body weight. Additionally, the size and weight of spleens were measured and used for the gene expression evaluation. CJLP 133 was used as the positive control [16].

### 2.4. Measurement of Skin Severity Score

The severity evaluation of HDM/DNCB-induced AD in mice was checked once a week as the skin severity score, according to a previously reported method by Jung et al. [17]. In brief, based on five symptoms including erythema/hemorrhage, edema, excoriation/erosion, scarring/dryness, and itching (10 min), the skin severity score was, respectively, scored as 0 (none), 1 (mild), 2 (moderate) and 3 (severe), and finally defined as the sum of the individual scores.

### 2.5. Measurement of Transepidermal Water Loss (TEWL)

To investigate the effect of SHE on the dorsal back skin dryness of HDM/DNCB-stimulated NC/Nga mice, we measured the value of TEWL on the dorsal skin of mice, according to the slightly modified method indicated in the previous study [18]. Temperature and humidity was, respectively, maintained at approximately 21 ± 2 °C and 50~55%. The value of TEWL was, respectively, monitored using the Tewameter TM300 system (Courage& Khazaka, Köln Germany), once a week for 5 weeks.

### 2.6. Measurement of Ear Skin Edema

Next, we confirmed the effect of SHE on the ear skin edema of HDM/DNCB-stimulated mice. At the end of the experiment, the ear skin thickness of all mice was checked with a digital caliper (Hornady, GI, USA), before the dissection.

### 2.7. Histological Observation of Dorsal Back Skin and Ear

At the dissection day, the dorsal back skin and ear tissues were fixed with 10% formalin, embedded in paraffin for the histological evaluation. The tissue sections were stained with hematoxylin and eosin for evaluation of hyperkeratosis and epidermal hyperplasia. In addition, the sections were stained with toluidine blue and Congo red, to detect the infiltration of mast cell and eosinophils in the dorsal back skin and ear, respectively [19]. The stained sections were examined with an Olympus AX80T microscope (Olympus, Tokyo, Japan), and the images were captured with a DP controller digital camera (DP71; Olympus, Tokyo, Japan).

### 2.8. Detection of Cytokine and Chemokine Expression in Dorsal Back Skin and Spleen

To detect the effect of SHE on the expression levels of cytokines and chemokines in dorsal AD skin and spleen of mice, reverse transcription–polymerase chain reaction (RT–PCR) was performed. Total RNA isolated from the dorsal back skin and spleen tissues by the Trizol reagent was used for the synthesis of each cDNA and used as a PCR product. PCR conditions were set for 5 min, denaturing at 94 °C, 1 min annealing at 55–60 °C, and a 20 min extension phase at 72 °C in a TaKaRa Thermal Cycler (TaKaRa Bio Inc., Otsu, Japan), and carried out for 35 cycles. Obtained PCR products were electrophoresed on 1.5% ethidium bromide/agarose gels and visualized under UV transillumination (Vilber Lourmat, Marne la Uallee, France). The nucleotide sequence of the primers used for PCR is indicated in Table 1 [19].

### 2.9. Measurement of Total Serum IgG_1_ and IgG_2a_

The serum levels of IgG_1_ and IgG_2a_ were separately measured using ELISA kits. In brief, the serum obtained from the mice was added to the microplate wells, specific to the coated antigen. Captured antibodies were incubated with serum samples, at room temperature for 1 h. Then, the microplates were blocked by blocking solutions. After blocking, the microplates were washed and treated with horseradish peroxidase (HRP) detection antibody. After incubation, the 3,3′,5,5′-tetramethylbenzidine (TMB) substrate was added to the microplates. The absorbance of each microplate was measured at 450 nm wavelength. The IgG_1_ and IgG_2a_ concentrations in each serum sample were estimated relative to the provided standard, following manufacturer’s instructions.

### 2.10. Measurement of Cyokines Production in Serum

Blood samples were drawn from mice, then it was centrifuged at 4000 rpm for 10 min, and the serum was collected. The concentrations of cytokines (IL-4, IL-5, IL-13, Eotaxin, and RANTES) in the serum were measured by the ELISA kit (Bio-Plex Reagent Kit, Hercules, CA, USA), according to the instructions of manufacturer.

### 2.11. Statistics

All experimental values are expressed as the mean ± SD of three determinations. Differences between the means of each group were assessed by one-way analysis of variance, followed by Duncan’s test using PASW statistics 21.0 software (SPSS, Chicago, IL, USA). A *p*-value < 0.05 was considered to be statistically significant.

## 3. Results

### 3.1. Oral Administration of SHE Improved the AD Symptoms in HDM/DNCB-Stimulated NC/Nga Mice

**Figure 2:**
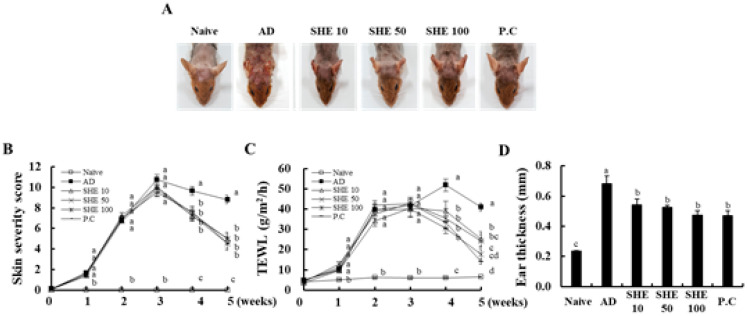
Suppression of HDM/DNCB-induced AD by oral administration of SHE (10, 50, and 100 mg/kg). (**A**) Comparing photographs of HDM/DNCB-induced skin lesions in NC/Nga mice after repeated oral administration of SHE. (**B**) Comparison of clinical skin severity scores of skin symptoms for 5 weeks after sensitization. (**C**) Comparison of TEWL value for 5 weeks after sensitization. (**D**) Comparison of ear thickness on day 35. Values were the mean ± SD of triplicate experiments. ^a–d^ Values with different alphabets were significantly different at *p* < 0.05 (*n* = 8).

To induce AD skin lesions, HDM/DNCB was repeatedly applied onto the dorsal surface of NC/Nga mice. As shown in Figure 2, during the first week, HDM/DNCB-stimulated mice showed mild dermatitis scores of 1.6 ± 0.2, on average. After 2 weeks, the AD-like skin lesions rapidly increased up to 7.0 ± 0.3, on average. In 3 weeks, the skin became thick and severe erythema, edema, hemorrhage, and erosion were observed in HDM/DNCB-stimulated mice groups. However, the oral administration of SHE significantly decreased AD-like skin lesions, compared to AD seen at 2 weeks after the first administration. These results showed similar activity with the P.C groups (Figure 2A,B).

### 3.2. Oral Administration of SHE Decreased the Dryness of the AD Dorsal Skin in the HDM/DNCB-Stimulated NC/Nga Mice

To evaluate the skin drying, the transepidermal water loss (TEWL) on the mice, dorsal skin was measured. In HDM/DNCB-stimulated mice, the value increased to >40 g/m^2^/h by 2 weeks, and an almost constant value was observed in the period that followed. The TEWL level was significantly inhibited upon the oral administration of SHE, throughout the experimental period. Especially, SHE 100 mg/kg showed the most effective activity when <20 g/m^2^/h, at 5 weeks (Figure 2C).

### 3.3. Oral Administration of SHE Reduced the Ear Edema in HDM/DNCB-Stimulated NC/Nga Mice

As illustrated in Figure 2D, HDM/DNCB-stimulation significantly increased the ear thickness of mice, whereas the administration of SHE dose-dependently decreased ear thickness, similar to the P.C.

### 3.4. Oral Administration of SHE Decreased the Serum IgG_1_ and IgG_2a_ levels in HDM/DNCB-Stimulated NC/Nga Mice

**Figure 3:**
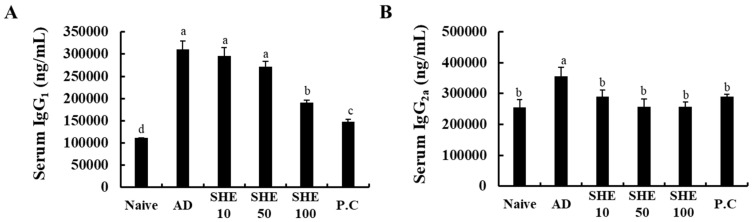
Evaluation of the serum levels of immunoglobulins in HDM/DNCB-induced mice. (**A**) Serum IgG_1_; and (**B**) IgG_2a_ levels of HDM/DNCB-induced mice, orally administered with SHE. Experiments were performed in triplicate, and the data were expressed as mean ± SD. ^a–d^ Values with different alphabets were significantly different at *p* < 0.05 (*n* = 8).

According to the present results, HDM/DNCB stimulation caused a significant increase in serum IgG_1_ and IgG_2a_ levels (Figure 3A,B). Interestingly the oral administration of SHE significantly ameliorated the serum IgG_1_ and IgG_2a_ levels, dose-dependently, to a level similar to the P.C, CJLP 133. These results indicated that the oral administration of SHE could effectively alleviate HDM/DNCB-induced AD-like skin lesions by suppressing the serum IgG_1_ and IgG_2a_ levels.

### 3.5. Oral Administration of SHE Inhibited the Hyperkeratosis and Epidermal Hyperplasia with the Reduction of the Infiltrated Mast Cells and Eosinophils in AD Dorsal skin and Ear Lesions of HDM/DNCB-Stimulated NC/Nga Mice

**Figure 4:**
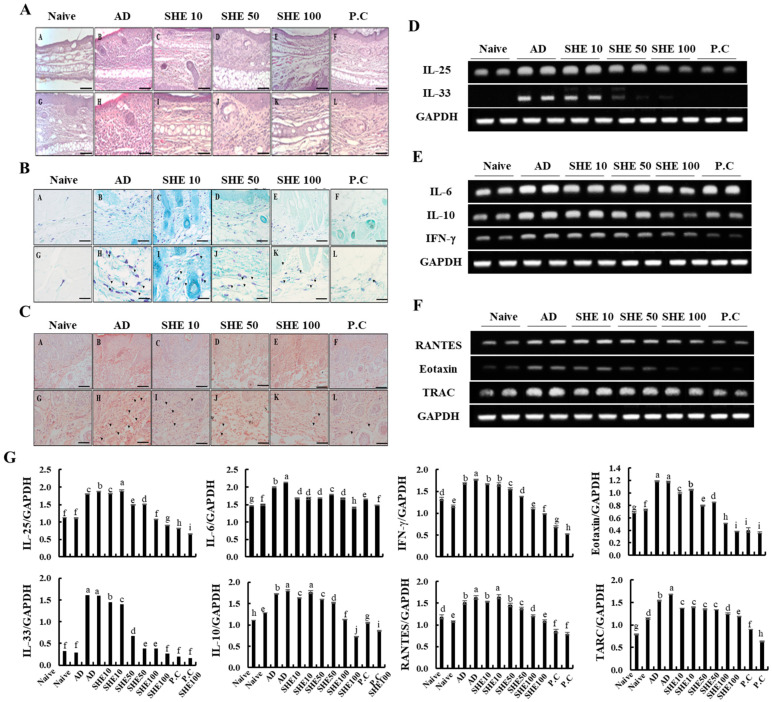
Results of hematoxylin and eosin-stained tissue (scale bar: 200 µm) and regulation of allergy-mediated mRNA expression levels of cytokines and chemokines expression in skin tissues of HDM/DNCB-induced mice. (**A**) Comparison of the histopathology of dorsal skin lesions after repeated oral administration of SHE on hematoxylin and eosin- stained tissues, (**B**) toluidine blue-stained skin, (**C**) congo-red stained skin, (**D**) regulation of SHE on atopy early stimulation factors (IL-25, IL-33), (**E**) regulation of SHE on allergic mediated cytokines (IL-6, IL-10, and IFN-γ), and (**F**) regulation of SHE on allergic mediated chemokines (RANTES, Eotaxin, and TRAC). Densitometry analysis (**G**). Experiments were performed in triplicates, to confirm repeatability. ^a–j^ Values with different alphabets were significantly different at *p* < 0.05 (*n* = 8).

As shown in Figure 4 and Figure S1, HDM/DNCB stimulation aggravated inflammatory changes like hyperkeratosis and epidermal hyperplasia. SHE treatment attenuated the increased skin thickness by HDM/DNCB, upon repeated induction, which was comparable to the P.C group. Based on histological changes in HDM/DNCB-stimulated dorsal skin and ear, it was evident that the increased number of dermal cells were inflammation mediator cells, including mast cells and eosinophils (Figure 4B,C). Additionally, the number of total and degranulated mast cells and eosinophils was much lower than those in the HDM/DNCB-stimulated groups.

### 3.6. Oral Administration of SHE Downregulated the mRNA Expression Levels of Cytokines and Chemokines in the AD Dorsal Skin and the Spleen of the HDM/DNCB-Stimulated NC/Nga Mice

**Figure 5:**
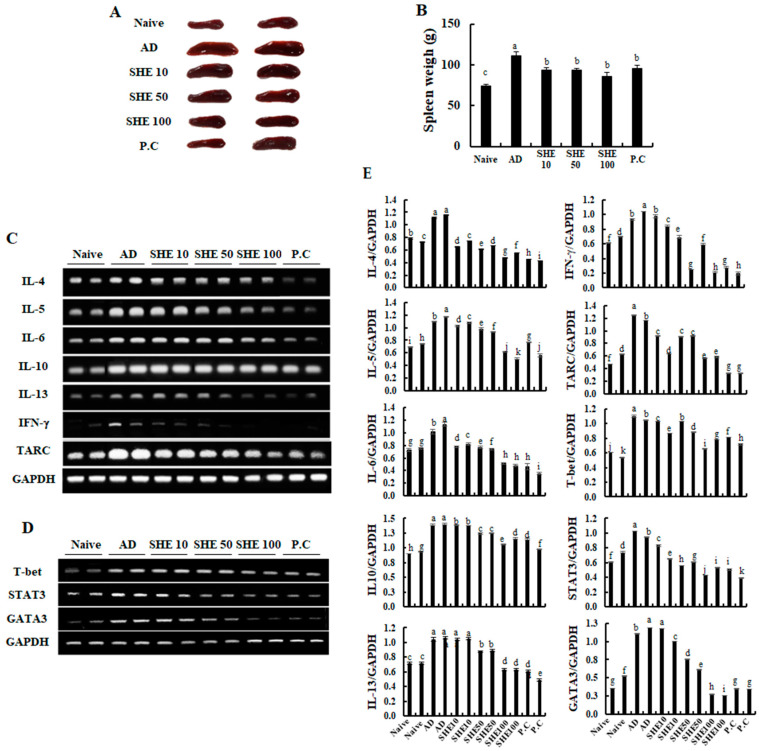
Changes observed in the spleen of HDM/DNCB-induced mice by SHE treatment. (**A**,**B**) Comparison of spleen size, (**C**) the mRNA expression levels of cytokines (IL-4, IL-5, IL-6, IL-10, IL-13, and IFN-γ) and chemokine (TARC), and (**D**) the mRNA expression levels of T-bet, STAT-3, and GATA-3. Densitometry analysis (**E**). Experiments were performed in triplicates, and the data were expressed as mean ± SD. ^a–k^ Values with different alphabets were significantly different at *p* < 0.05 (*n* = 8).

Effects of SHE on mRNA expression levels of cytokines and chemokines was examined, which are crucial markers responsible for the pathogenesis of AD, in mice dorsal skin. As shown in Figure 4D, HDM/DNCB stimulation markedly increased the mRNA expression levels of AD-related cytokines, such as IL-25 and IL-33, compared to naive groups. However, the oral administration of SHE could reduce their expression levels. Additionally, the administration of SHE inhibited the mRNA expression levels of allergy mediating cytokines, IL-6, IL-10, and IFN-γ in HDM/DNCB stimulated mice (Figure 4E). Moreover, the administration of SHE suppressed the expression levels of chemokines RANTES and TARC, which was increased by HDM/DNCB stimulation (Figure 4F). Upon HDM/DNCB stimulation, changes were observed in the spleen size and specific splenic cytokines and chemokine mRNA expression levels. As Figure 5A and B illustrate, the spleen size and weight were increased by HDM/DNCB. However, oral administration of SHE dose-dependently decreased the spleen size, whereas at 100 mg/kg SHE concentration, the spleen size was similar to the CJLP 133 treatment group. According to Figure 5C,D, HDM/DNCB stimulation upregulated the expression levels of T-bet, STAT3, and GATA3 and other allergy mediating cytokines (IL-4, IL-5, IL-10, IL-13, and IFN-γ) and chemokine (TARC). SHE treatment dose-dependently attenuated mRNA expression levels of cytokines and chemokine aggravated by HDM/DNCB.

### 3.7. Oral Administration of SHE Suppressed the Production of Cytokines in the HDM/DNCB-Stimulated NC/Nga Mice Serum

**Figure 6:**
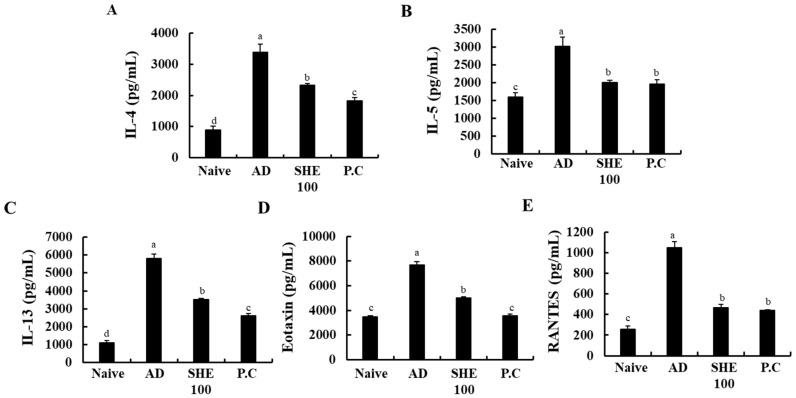
The production of IL-4 (**A**), IL-5 (**B**), IL-13 (**C**), Eotaxin (**D**), and RANTES (**E**) in HDM/DNCB-stimulated mice serum. Experiments were performed in triplicates, and the data were expressed as mean ± SD. a-dValues with different alphabets were significantly different at *p* < 0.05 (*n* = 8).

Effects of SHE on production of cytokines was examined, which are crucial markers responsible for the pathogenesis of AD, in mice serum. As shown in Figure 6, HDM/DNCB stimulation markedly increased the production of AD-related cytokines, such as IL-4, IL-5, IL-13, Eotaxin, and RANTES, compared to the naive groups. Interestingly, the oral administration of SHE significantly suppressed their production.

## 4. Discussion

The present study disclosed the investigation of the effects of SHE on AD-like skin lesions and abnormal immune responses in HDM/DNCB-induced AD NC/Nga mice model. AD is one of the chronic inflammatory skin diseases characterized by a hypersensitive skin that can be induced by environmental factors, immunological, and genetic factors [20]. The symptoms of AD include skin itching, swelling, erythema, dried and cracked skin, and abnormal immune response, such as the overproduced IgE and the overactivation of immune cells like basophils, eosinophils, and mast cell, as well as T-helper (Th) cells [21].

Early studies indicated that HDM, mold, pollens of birch, grass, and ragweed cause exacerbation of AD [22]. Among them, HDM and DNCB are frequently used to induce AD in mice models, as they provide results with higher reproducibility and repeatability [23]. The NC/Nga mouse model is widely used for the investigation of drugs or food candidates for the treatment of AD, and is considered to be a representative in vivo model mimicking human AD symptoms [3,5,18,19]. Here, we treated both HDM and DNCB as co-inducers of AD into NC/Nga mice, according to the modified method described by Tanaka (2015) [24].

Over the years, numerous steroids like topical agents were used to treat AD [25]. However, these drugs cause numerous side effects like hormone imbalance, bone loss, and heart diseases [25,26,27]. Thus, the exploration of safer alternatives that satisfy consumer’s demand while minimizing side effects, compared to existing drugs, received increased attention [28].

Many researchers focus on exploring the natural materials that can improve these abnormal immune responses. CJLP 133 is one of the kimchi-derived lactic acid bacteria that is now developed as a functional food. The present study focused on the development of functional food that can improve immune responses similar to that of CJLP 133^11^. Our previous study disclosed the anti-allergy effects of SHE that reduce the IgE/BSA-induced mast activation. Hence, further studies were undertaken to investigate the effect of SHE against AD in mice model ^11^. Present outcomes revealed that the oral administration of SHE improved the AD symptoms, decreasing skin severity score, including erythema, hemorrhage, edema, excoriation, erosion, scarring, dryness, and itching. Especially, the skin dryness in AD dorsal skin lesion, following the increased TEWL was significantly reduced by the administration of SHE in the HDM/DNCB-stimulated NC/Nga mice. Thus, with repeatable observations, it was confirmed that SHE is effective in reducing the loss of skin moisturization resulting from HDM/DNCB-induced AD.

The dermal infiltration of inflammatory cells, such as mast cells and eosinophils, is a major histopathological incident of AD [29]. Activated mast cells release various substances that mediate events seen during AD, which eventually activate eosinophils [30]. The events cause vascular leakage, resulting in edema formation. The decrease of edematous ear thickness by SHE treatment, could be attributed to the reduction of immune cell infiltration and activation. Hematoxylin and eosin-stained sections of skin and ear tissues from NC/Nga mice were examined for hyperkeratosis and epidermal hyperplasia. Aggravated inflammatory responses upon HDM/DNCB stimulation, such as hyperkeratosis and epidermal hyperplasia, were effectively reduced following SHE treatment, as well as skin thickness, as compared to the P.C group. Based on histological changes in HDM/DNCB-stimulated dorsal skin and ear, it was apparent that the increased number of dermal cells were inflammation mediator cells, including mast cells and eosinophils. Additionally, the number of total and degranulated mast cells and eosinophils was much lower than those in the HDM/DNCB-stimulated groups. Thus, these results indicated that the oral administration of SHE decreased the hyperkeratosis and epidermal hyperplasia in skin and ear by suppressing the infiltration of inflammatory cells.

IL-25 and IL-33 are epithelial cell-derived cytokines that lead to Th2 cytokine-mediated allergy responses, either directly by the Th2 cell or indirectly via dendritic cell activation. Additionally, it affects the activation of other mediators, including mast cells, eosinophils, and basophils [31]. IL-25 was constitutively expressed in epithelial cells and was released by HDM stimulation and proteases, like trypsin and papain, whereas, it affected the exacerbation of allergic diseases [32]. Additionally, IL-33 is crucial for the development of HDM-induced allergic and anaphylaxis responses, and activation of T-cells. T-cells are crucial for the mediation of most immune diseases. Generally, skin lesions seen in AD features T-cell infiltration and production of both Th1 and Th2 type cytokine and chemokines derived from the T-cells [32]. The underlying mechanisms responsible for the chronicity of allergic inflammation remain unclear, but generally, it is well-known that Th2 cytokines like interleukin (IL)-4, IL-5, IL-6, IL-10, and IL 13 play essential roles in the development of AD [33]. Based on a recent study, IFN-γ of Th1 cytokines are excessively expressed in patients during skin lesion of AD, which is found to play a crucial role in the acute responses of AD [21]. IL-6 is a pro-inflammatory cytokine that affects B-cell differentiation into plasma cells, which in turn produce excessive IgE in serum. According to a previous study [34], a high level of IL-10 was detected in the serum of AD-induced mice, and it activated the production of other Th2 cell-induced cytokines and chemokines, as well as Th1 cells [35].

An increase in serum IgG_1_ and IgG_2a_ levels is a key of AD. IgG1 and IgG2a are closely related to the production of Th1 and Th2 cytokines, and directly affect the production of IgE. Fundamentally, the Th1/Th2 imbalance results in the segregation of IgG1 and IgG2a immunoglobulin isotypes, and affects the skin tissue deterioration seen during AD [35,36]. Present results indicated a significant increase in serum IgG_1_ and IgG_2a_ levels in the HDM/DNCB-stimulated group. Oral SHE administration significantly ameliorated the serum IgG_1_ and IgG_2a_ levels, to a level comparable with P.C and CJLP 133, thus, indicating that oral administration of SHE could effectively alleviate HDM/DNCB-induced AD-like skin lesions by suppressing serum IgG_1_ and IgG_2a_ levels (Figure 2).

The mRNA expression levels assessed during the present study indicated a marked increase in AD-related early stimulation cytokines like IL-25 and IL-33, upon HDM/DNCB stimulation as compared to the naive groups. However, effective reduction of their expression levels upon oral administration of SHE suggests its effectiveness in suppressing the early effects of AD (Figure 4D). Levels of allergy mediating cytokines, IL-6, IL-10, and IFN-γ, as well as chemokines RANTES and TARC were also reduced upon SHE administration (Figure 4E). Suppression of allergy mediated cytokine and chemokines production reinforce the SHE’s anti-allergic functionalities.

AD not only induce skin lesions, but also affects internal tissues and organs like the spleen, bone marrows, and lymph nodes [37]. Among the above-mentioned organs, the spleen is the most affected by AD. Activation of T and B cells causes the proliferation of splenic cells, which causes spleen enlargement [38]. According to recent studies, cytokines and chemokines of Th1 and Th2 cells, such as IL-4, IL-5, CCR3, IL-12, and Eotaxin levels were significantly increased in the spleen of HDM/DNCB-stimulated mice [17,37]. We came across similar observations with an apparent increase in spleen size and volume, and an increase of certain splenic cytokines and chemokine mRNA expression in HDM/DNCB-stimulated mice (Figure 4). SHE treatment, dose-dependently reduced the spleen size and weight, as well as Th1 and Th2 cytokines and chemokines, similar to the CJLP 133 treatment group. The GATA-3, STAT-3, and T-bet are essential transcription factors for T-cell differentiation. Naive CD4^+^ T cells can differentiate into distinct effector types that tailor immune responses after antigen exposure. Upregulated GATA-3 and STAT-3 could direct T-cell differentiation to eosinophils, basophils, mast cells, and Th2 cells, whereas T-bet differentiates T-cells into Th1 cells [39]. In line with present observations, (Figure 4) HDM/DNCB stimulation upregulated the T-bet, STAT3, and GATA3 and other allergy mediating cytokines (IL-4, IL-5, IL-10, IL-13, and IFN-γ) and chemokine (TARC) expression levels, while these were dose-dependently downregulated following SHE treatment. Thus, suggesting the effectiveness of SHE oral-administration towards alleviating AD-related expression levels of various splenic cytokines and chemokines.

## 5. Conclusions

Collectively, the present observations suggest that SHE possess beneficial potentials that could ameliorate AD by inhibiting the development of eczematous lesions, by suppressing TEWL, serum IgG_1,_ and IgG_2a_ levels, hyperkeratosis, and epidermal hyperplasia, in the skin and ear by suppressing the infiltration of inflammatory cells in HDM/DNCB-induced AD NC/Nga mice models. With pre-clinical trials in place, SHE can be developed as a natural functional food supplement to counter AD.

## Figures and Tables

**Table 1:** RT–PCR primers.

Primers	Sequence
IL-4	Forward: ACG GAG ATG GAT GTG CCA AAC
	Reverse: AGC ACC TTG GAA GCC CTA CAG A
IL-5	Forward: TCA GCT GTG TCT GGG CCA CT
	Reverse: TTA TGA GTA GGG ACA GGA AGC CTC A
IL-6	Forward: CCA CTT CAC AAG TCG GAG GCT TA
	Reverse: GCA AGT GCA TCA TCG TTG TTC ATA C
IL-10	Forward: GAC CAG CTG GAC AAC ATA CTG CTA A
	Reverse: GAT AAG GCT TGG CAA CCC AAG TAA
IL-13	Forward: CAA TTG CAA TGC CAT CTA CAG GAC
	Reverse: CGA AAC AGT TGC TTT GTG TAG CTG A
IL-25	Forward: CTC AAC AGC AGG GCC ACT C
	Reverse: GTC TGT AGG CTG ACG CAG TGT G
IL-33	Forward: GAT GAG ATG TCT CGG CTG CTT G
	Reverse: AGC CGT TAC GGA TAT GGT GGT C
IFN-g	Forward: CGG CAC AGT CAT TGA AAG CCT A
	reverse: GGC ACC ACT AGT TGG TTG TCT TTG
TARC	Forward: TGA GGT CAC TTC AGA TGC TGC
	Reverse: ACC AAT CTG ATG GCC TTC TTC
RANTES	Forward: GGA GTA TTT CTA CAC CAG CAG CAA G
	Reverse: GGC TAG GAC TAG AGC AAG CAA TGA C
Eotaxin	Forward: AAC ATG GCG GGC TCT GCT AC
	Reverse: CCT GCC TTG GGA CAG ATG CT
STAT3	Forward: GA AGC CGA CCC AGG TGC
	Reverse: GT CAC GTC TCT GCA GCT TCT
T-bet	Forward: CG GTA CCA GAG CGG CAA GT
	Reverse: AG CCC CCT TGT TGT TGG TG
GATA3	Forward: TC TCA CTC TCG AGG CAG CAT GA
	Reverse: GGT ACC ATC TCG CCG CCA CAG
GAPDH	Forward: CAT CCG TAA AGA CCT CTA GCC AAC
	Reverse: ATG GAG CCA CCG ATC CAC A

## Supplementary Materials

The following are available online at https://www.mdpi.com/2072-6643/12/8/2482/s1. Figure S1: Comparison of the ear edema and histopathology of ear lesions in HDM/DNCB-induced AD mice. (A) Ear thickness, (B) comparison of the histopathology of ear lesions after repeated oral administration of SHE on hematoxylin and eosin-stained tissues, (C) toluidine-blue-stained skin, (D) congo-red stained skin. Experiments were performed in triplicates and the data were expressed as mean ± SE. Values with different alphabets were significantly different at *p* < 0.05, as analyzed by PASW statistics 21.0 software.
