# Supplementary material for: Oral Administration of Sargassum horneri Improves the HDM/DNCB-Induced Atopic Dermatitis in NC/Nga Mice
Source: Nutrients. 2020 Aug 18;12(8):2482. doi: 10.3390/nu12082482 (PMC7468899; doi:10.3390/nu12082482)
Supplement: Supplementary file 1 [file nutrients-12-02482-s001.pdf]

nutrients

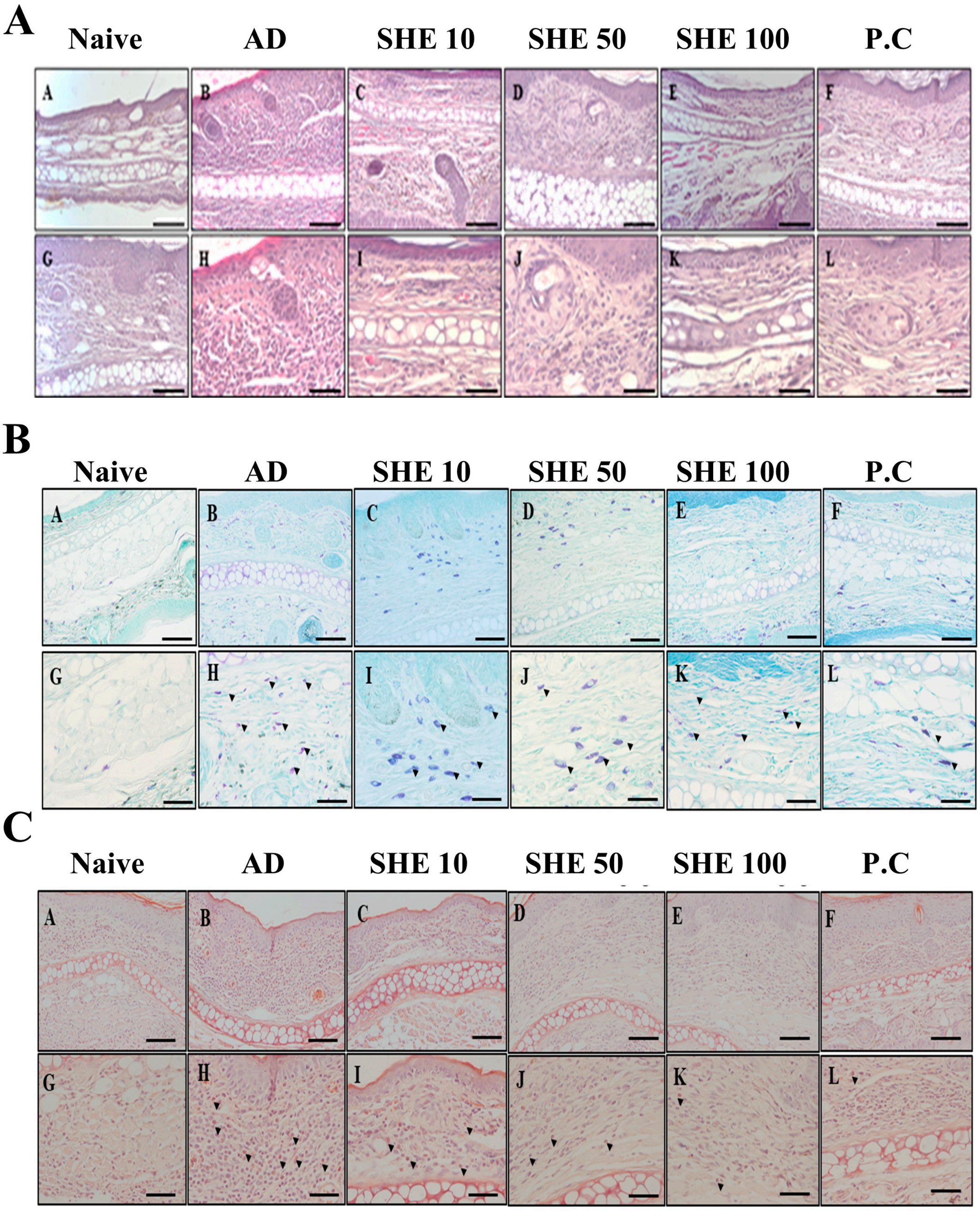

Figure S1. Comparison of the ear edema and histopathology of ear lesions in HDM/DNCB-induced AD mice. (A) Ear thickness, (B) comparison of the histopathology of ear lesions after repeated oral administration of SHE on hematoxylin and eosin-stained tissues, (C) toluidine-blue-stained skin, and (D) congo-red stained skin. Experiments were performed in triplicates and the data were expressed as mean ± SE. Values with different alphabets were significantly different at $p < 0.05$, as analyzed by PASW statistics 21.0 software.
